# Accounting for the environment as an economic asset: global progress and realizing the 2030 Agenda for Sustainable Development

**DOI:** 10.1007/s11625-015-0350-4

**Published:** 2015-12-12

**Authors:** Emma Terama, Ben Milligan, Rafael Jiménez-Aybar, Georgina M. Mace, Paul Ekins

**Affiliations:** 1 Institute for Sustainable Resources, University College London, 14 Upper Woburn Place, London, WC1H0NN UK; 2 Climate Change Programme, Finnish Environment Institute, 00251 Helsinki, Finland; 3 Centre for Law and Environment, University College London, London, WC1E 6BT UK; 4 GLOBE International, 1150 Brussels, Belgium; 5 Centre for Biodiversity and Environment Research, University College London, London, WC1E 6BT UK

**Keywords:** Natural capital accounting (NCA), SDGs, 2030 Agenda for Sustainable Development, Ecosystems, Sustainability, Policy, Cooperation

## Abstract

National and international efforts to develop natural capital accounts are proliferating. The newly agreed 2030 Agenda for Sustainable Development echoes these efforts. Continued cooperation is needed to overcome key scientific and policy challenges.

## Introduction

In September 2015, the United Nations General Assembly unanimously adopted a new Agenda for Sustainable Development (UN 2015). The Agenda features 17 Sustainable Development Goals (SDGs) and 169 associated targets, which UN member countries have committed to implement by 2030. An important feature of the Agenda is its clear recognition that social and economic development hinges on the sustainable management of the natural environment and its resources.

The term ‘natural capital’ is now widely used to describe components of the natural environment (e.g. minerals, fuels, animals and plants, ecosystems) that provide valuable goods or services (Millennium Ecosystem Assessment—MEA 2005; Kumar 2012; UK Natural Capital Committee—UK-NCC 2014; Mace et al. 2015). This characterization is appealing from a policy perspective because it enables nature to be treated like other valuable capital assets—i.e. as something that should be managed, valued and accounted for, and where policy or management interventions are necessary to avert or repair damage to the asset that may affect its ability to provide goods or services in the future (Milligan et al. 2014). Accounting for ecosystems as ‘assets’ can support policymaking and future action to realize the 2030 Agenda for Sustainable Development.

However, the use of natural capital as a monetary concept in policy processes has also attracted criticism, in particular from the academic community. For example, it is argued that the wording oversimplifies ecological complexity, marginalizes non-economic values of nature, conflicts with social and environmental justice, or facilitates nature’s ‘commodification’ or ‘privatization’ (Norgaard 2010; Gómez-Baggethun et al. 2010; Gómez-Baggethun and Ruiz-Pérez 2011; Matulis 2014). Here, we chose to characterize natural capital more broadly as a national, natural resource that needs to be fully accounted for to secure its benefits for present and future generations.

Scientific research has characterized, with increasing granularity and sophistication, the physical stock and flow of goods and services provided by nature and their fundamental contributions—many irreplaceable—to human well-being and development (MEA 2005). Efforts to quantify (1) the state and extent of the ecosystems and flows originating from these systems, and (2) the economic importance thereof at different spatial or temporal scales have proliferated (Kumar 2012; UK-NCC 2013; UNU-IHDP and UNEP 2012; Stiglitz et al. 2009; World Bank 2011).

Translating these bodies of knowledge into policy action is a critical global challenge in an era of increasing population, consumption and environmentally critical impacts. This process is well on its way, not least due to the recent agreement on the global SDGs. The physical stock of natural capital worldwide is being rapidly depleted, in some cases irreversibly (MEA 2005), and conventional approaches to measuring and managing economic activity do not adequately take this into account (Stiglitz et al. 2009). The status of natural assets is not, for example, captured comprehensively by accounting frameworks such as the widely used United National System of National Accounts (European Commission et al. 2009). Likewise, the most politically influential measure of national economic activity—the Gross Domestic Product (GDP)—does not, and was never intended to, account for the full value of the stock and flows from natural capital. Against this background there is an urgent need to implement more effective methods and measures for natural capital accounting to capture the portion of stock and flows from natural capital and ecosystem services that are currently not reflected in the standard economic accounts. These need to be embedded within relevant economic and environmental policies.

In this article, we highlight the status of efforts to address this need at international level—through development of international policy frameworks, strategies and standards, and at a national level through legislative and policy reform in a diverse group of 21 countries. We also identify key challenges that impede further progress and discuss how these might be addressed.

## Natural capital and accounting

**Fig. 1:**
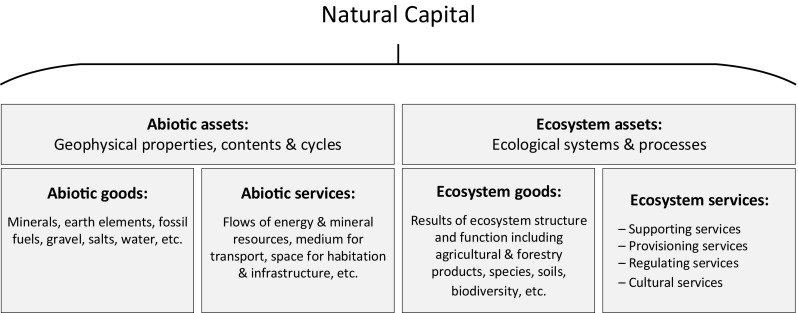
Component stocks of natural capital and associated flows

Natural capital is a diverse asset class that has been categorized or conceptualized in a variety of complex ways (MEA 2005; Kumar 2012; UK-NCC 2014; Milligan et al. 2014; UNU-IHDP and UNEP 2012; Stiglitz et al. 2009; World Bank 2011). Figure 1 presents a basic typology of natural capital stocks, and the associated flows of goods and services. It distinguishes between ‘abiotic capital’—the geophysical properties and contents of the Earth, including geophysical cycles—and ‘ecosystem capital’—the dynamic complexes of biotic communities and their non-living environment, including water and soils that interact with each other as a functional unit (MEA 2005).

National natural capital accounting is designed to measure the status of country-level natural capital in terms of its contribution to national wealth. The ultimate goal is to safeguard the stocks of critical natural capital that contribute to human well-being (Mace et al. 2015). It supplements conventional economic activity measurements such as GDP with the aim to: (1) obtain, process and communicate scientific information concerning the status of natural capital and associated trends; (2) apply valuation or costing methodologies to identify the economic implications of natural capital, including how it contributes to wealth and well-being; (3) process and communicate the results.

## International frameworks, strategies and standards

In recent years, efforts have proliferated to develop international frameworks, strategies and standards concerning natural capital accounting (Milligan et al. 2014). Relevant efforts fall into three broad and interrelated categories: legal and political commitments, development of standardized technical methods, and capacity and knowledge building partnerships. These are discussed below.

First, legal and political commitments relating to natural capital accounting have been established through a wide variety of international instruments, including under the auspices of several multilateral environmental agreements. For example: The 193 States Parties to the 1992 Convention on Biological Diversity (CBD) have committed, in one of the 2011–2020 Aichi Biodiversity Targets, to integrate ‘biodiversity values’ into their national accounting. In October 2014, the CBD meeting of the conference of the parties (COP 12) produced the publication “Ecosystem Natural Capital Accounting (ENCA): A Quick Start Package” which is currently under experimental implementation. The “Agenda 21” outcome document of the 1992 ‘Rio’ UN Conference on Sustainable Development explicitly characterizes natural and biological resources as capital assets, and sets out action plans for ‘establishing systems for integrated environmental and economic accounting’. “The Future We Want” outcome document of the subsequent 2012 ‘Rio+20’ Conference reaffirms international commitments to Agenda 21 and the Aichi Biodiversity Targets, recognizes the importance of ecosystem services as ‘critical foundations for sustainable development and well-being’ and requests the UN Statistical Commission to launch a programme of work concerning ‘broader measures of progress to complement GDP’.

From 2015 onwards the 2030 Agenda (UN 2015) is an important focal point for such cooperative efforts, especially those focused on building political awareness and will. The Agenda preamble—including the set of SDGs—entitled ‘Transforming our world’ was adopted by UN member States at the September 2015 summit. It is expected to fundamentally influence international politics and funding for sustainable development, thereby shaping future policy efforts and momentum to account for natural capital.

As a result of the intensive intergovernmental negotiations leading to the 2030 Agenda that commenced back in January 2015, a considerable status is attributed to natural capital and natural capital accounting within the Agenda and formative SDGs: ‘32. We recognise that social and economic development depends on the sustainable management of our planet’s natural resources.’ For the first time, the UN General Assembly Open Working Group recognizes the insufficiency of currently available measures: ‘48. […]We are committed to developing broader measures of progress to complement gross domestic product (GDP).’ This new wording represents the outcome of core negotiations and discussions in stakeholder consultations via, e.g. the UN Sustainable Development Knowledge Platform.

Natural capital accounting features in the targets accompanying the SDGs, in similar fashion to the existing international legal and political commitments discussed above, including the CBD, associated Aichi Targets, and outcome documents of the Rio and Rio+20 Conferences. Goal 15 (concerning terrestrial ecosystems, forests, desertification, land degradation and biodiversity loss) is accompanied by a target to ‘By 2020, integrate ecosystem and biodiversity values into national and local planning, development processes, poverty reduction strategies and accounts (15.9)’. It is unfortunate that this terrestrial ecosystems-related target is not complemented by an equivalent for Goal 14 on ocean ecosystems and marine resources (UN 2015).

Second, legal and political commitments have stimulated efforts to develop standardized technical methods for natural capital accounting. Noteworthy are efforts by the World Bank and the UN, as well as the Inclusive Wealth Project. The UN System of Environmental–Economic Accounting (SEEA) was first published in 1993 by the UN Statistical Commission to implement commitments agreed at the 1992 Rio Conference. Significant revisions were published in 2003 and 2012, and in 2013 the SEEA Experimental Ecosystem Accounting (SEEA-EEA: European Commission et al. 2013), for addressing the living, biotic components of natural capital. The SEEA is designed to supplement the System of National Accounts, and contains internationally agreed standards for producing comparable statistics concerning the environment and its relationship with the economy (UN et al. 2014). The World Bank (2011) data catalogue hosts the adjusted net saving and non-renewable resource rent indicators for the period 1970–2008. Adjusted net saving measures the rate of saving in an economy, after taking into account investments in human capital and depletion of natural resources, in an attempt to assess the sustainability of that economy. Also the Inclusive Wealth Project has produced a report on human capital in addition to the 2012 report on natural capital with the accompanying Inclusive Wealth Index (UNU–IHDP and UNEP 2012).

Third, implementation of SEEA and other relevant standards at a national level is now supported by several capacity and knowledge building partnerships. Since 2010 the World Bank has coordinated the Partnership for Wealth Accounting and the Valuation of Ecosystem Services (WAVES), which involves partners including UN Agencies, civil society representatives and national governments. WAVES provides technical support to several ‘Core Implementing Countries’ including: Botswana, Colombia, Costa Rica, Guatemala, Indonesia, Madagascar, the Philippines and Rwanda, with the number of countries due to expand shortly. Other efforts include The Economics of Ecosystems and Biodiversity Initiative (TEEB)—established in 2007 which focuses on knowledge synthesis and capacity building, and the Global Legislators Organization’s Natural Capital Initiative—established in 2012, focusing on knowledge sharing concerning legal and policy aspects of natural capital accounting. Also Goal 17 of the Agenda for Sustainable Development now involves implementation and global partnership, with targets on finances, technology and capacity-building aspects (UN 2015).

## National legislative and policy reform

The international efforts mentioned above are accompanied by—and inter-linked with—concerted national level efforts to (1) develop legal and policy frameworks for natural capital accounting, and (2) link these frameworks with broader policies for managing natural assets. Working in partnership with national contributors (including members of parliament, government officials, or subject matter experts) we reviewed legislative and policy reforms concerning natural capital accounting in a group of 21 countries (Milligan et al. 2014). The countries included have diverse national income levels (5 high-income, 14 middle-income and 2 low-income countries) and a wide geographical spread (7 African, 4 Asian, 4 European, 4 North American and 2 South American countries).

The study collated evidence from national and international sources, as well as national expert informants about the status of policy, objectives, and examples of practice regarding natural capital accounting. A noteworthy feature of the reviewed national policy reforms is their diversity. Countries have responded in very different ways to the calls for natural capital accounting, including methods, standards, institutional structures, legal requirements, and broader policy objectives associated (Milligan et al. 2014). Among the countries reviewed here, four broad categories were identified: (1) countries with national strategies or policy commitments to develop natural capital accounts in the near future (e.g. India, Democratic Republic of the Congo); (2) countries undertaking active investigation and pilots to assess the feasibility of different options for natural capital accounting (e.g. France, Georgia, the Philippines, Rwanda); (3) countries in which natural capital accounting activities are already taking place, supported by a legal or policy framework (e.g. Colombia, Ghana, Mexico, UK); (4) countries where natural capital accounts are being used to inform politics and government decision-making concerning natural assets on an ongoing basis (e.g. Costa Rica, Guatemala and Peru).

Another feature of national reforms of existing accounting and policy concerning natural capital accounting and sustainability is their reliance on continued cooperation and diverse forms of support. This has an international dimension: accounting standards such as the SEEA, commitments such as the CBD, and capacity and knowledge building partnerships such as WAVES and TEEB all play a role in supporting national reforms. National approaches also entail efforts involving various parts of government and diverse stakeholders, including local communities and the private sector. The exact make-up of these partnerships and their coordination and governance varies greatly by country: Botswana has water accounts developed in partnership with WAVES, and is member of the Gaborone Declaration (2012) pledging to “integrate the value of nature into their national policies and programmes, recognizing that nature is needed for economic growth and sustainability” together with nine other African countries; Canada has detailed multi-sector accounting frameworks with Statistic Canada leading the compilation of natural capital accounts, whilst measurement and data is being provided by federal, provincial and territorial governments; China has set up compensation mechanisms for ecological restoration and management of forests, grasslands and wetlands, involving the mining sector as well as local farmers. While these collaborative efforts at both national and international scale have proliferated, there seems to be a great demand for guidance and support in the area of natural capital accounting, and sustained co-coordination of future activities is needed between the currently diverse range of actors.

## Addressing key challenges

**Fig. 2:**
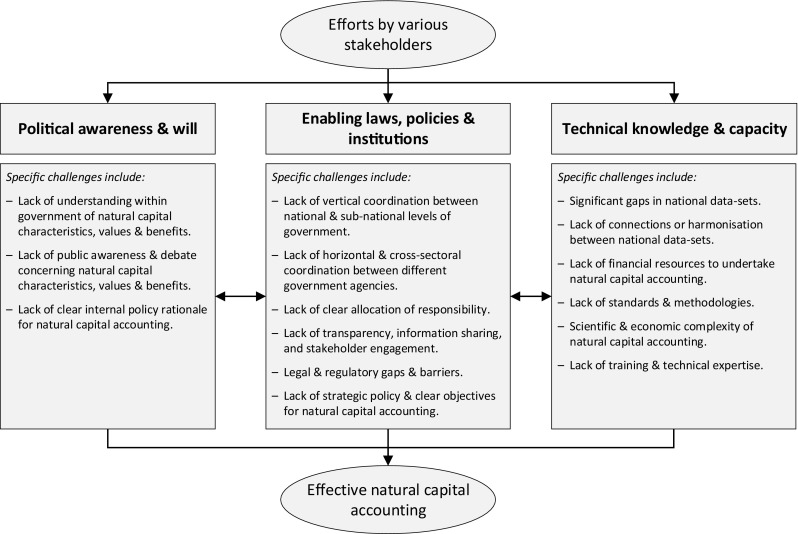
Natural capital accounting—key challenges to national implementation

Natural capital accounting is a complex undertaking that in most countries is either absent or relatively new. Figure 2 divides national challenges identified by the 21 country participants into three broad groups: Political awareness and will; Enabling laws, policies and institutions; and Technical knowledge and capacity. All of the national partners we have worked with highlight that further progress is complicated by significant challenges, including those identified in Fig. 2. Well-resourced cooperation to develop context-appropriate solutions for these challenges should be a political priority—for national and international policymakers, and other decision makers capable of influencing policy development.

The new Sustainable Development Goals and targets will come into effect on 1 January 2016, and will guide the decisions we take over the next fifteen years. The specific aim to ‘By 2030, achieve the sustainable management and efficient use of natural resources (12.2)’ is a powerful signal for policymakers for taking immediate action to ensure speedy progress for realizing the agreed Agenda. Natural capital accounting can be seen as part of the Agenda as an indicator, providing a means to achieve measurable outcomes towards particular SDGs and accompanying targets (UNSDSN 2015, UN 2015). The Goals will have various implications: broad targets such as 12.2 will focus attention on policy reform in a manner that accommodates continued diversity of national approaches to natural capital accounting whilst Goal 17 recognizes explicitly the need to support countries with limited resources: both financial and in capability. Recognizing accounting (15.9) as a necessary means to measuring progress in sustainable development will focus attention on the challenging task of developing standardized and comparable national accounts to fully operationalize natural capital as an element required for success in sustainability.

Ultimately, getting serious about sustainable development requires that national governments measure (also in non-monetary ways) and monitor natural assets at least as carefully as they do other physical assets and income flows. Creating natural asset inventories, mapping the dependence of national well-being on local ecosystems and abiotic goods, and assessing their status and associated trends provide precious information for policy makers by revealing the case for sustainable development. Systemic accounting for natural capital complements this information by providing a crucial, practical tool for operationalizing economic development within environmental limits, and informing efforts to implement national and global visions for a more sustainable future. Finally, we propose more research into the area of natural capital accounting as a way to promote global sustainable development and realizing the 2030 Agenda.
